# Kombucha Fermentation of Olympus Mountain Tea (*Sideritis scardica*) Sweetened with Thyme Honey: Physicochemical Analysis and Evaluation of Functional Properties

**DOI:** 10.3390/foods12183496

**Published:** 2023-09-20

**Authors:** Ioannis Geraris Kartelias, Haralabos Christos Karantonis, Efstathios Giaouris, Ioannis Panagiotakopoulos, Constantina Nasopoulou

**Affiliations:** 1 Laboratory of Food Chemistry and of Technology and Quality of Animal Origin Food, Department of Food Science and Nutrition, School of the Environment, University of the Aegean, 81400 Myrina, Greece; fnsd21002@fns.aegean.gr (I.G.K.); fnsd21009@fns.aegean.gr (I.P.); knasopoulou@aegean.gr (C.N.); 2 Laboratory of Food Microbiology and Hygiene, Department of Food Science and Nutrition, School of the Environment, University of the Aegean, 81400 Myrina, Greece; stagiaouris@aegean.gr

**Keywords:** kombucha, *Sideritis scardica*, thyme honey, antioxidant, bioactivities

## Abstract

This study implemented kombucha fermentation of Olympus Mountain tea (*Sideritis scardica*) sweetened with honey (OMTWH) in order to investigate the potential for producing a novel beverage with functional properties. The increase in the total count of bacteria and yeast suggests that the OMTWH acts as a viable substrate for supporting the proliferation of the microorganisms of the Kombucha symbiotic culture. The fermentation resulted in a reduction in pH and increased total titratable acidity. After fermentation, a statistically significant increase in the vitamins C, B1, B2, B6, B7, and B12 content was observed (*p* < 0.05). Total phenolics and antioxidant activity of the fermented beverage was significantly enhanced, as assessed by the method of Folin–Ciocalteu and ABTS assay, respectively. Results revealed that OMTWH had a potent inhibitory activity of α-amylase, α-glucosidase, acetylcholinesterase, and butyrylcholinesterase; OMTWH fermented with a kombucha consortium exhibited even higher inhibition. Hence, the process of kombucha fermentation can convert OMTWH into a novel beverage with enhanced functional properties.

## 1. Introduction

The consumption of certain beverages for the prevention of chronic diseases and maintaining good health has now been recognized worldwide. The scientific community and consumers are keen to study and consume bioactive compound-rich beverages. Despite the availability of various beverages in the market with functional properties, there is a need to search for new combinations and ratios of raw materials to improve the bioactivity in the final products. Moreover, consumer lifestyles have changed in recent years and will be influenced by globalization, economic and social changes, economic growth, rapid advances in food science, food and lifestyle choices, and religious restrictions [1,2,3].

Beverages are an excellent vector for the transfer of nutrients and bioactive compounds in the body, as well as facilitating bioavailability. Bioactive compounds, such as phytochemicals (e.g., phytoestrogens, phenolic compounds, flavonoids, and carotenoids), dietary fiber, vitamins, fatty acids, probiotics, and minerals, can be incorporated into beverages. The presence of these compounds provides the prospect of using food as a valuable element in strategies to disease curing, particularly in the early stages of diseases [4,5,6]. This is in line with the increasing trend in the consumption of functional drinks [7] due to their nutritional content [8].

Functional beverages can be classified according to the basic ingredients: milk, fruit and vegetables, fruit and legumes, cereals, coffee, and tea. These beverages’ functional characteristics cover different needs and lifestyles, such as providing energy, inhibiting aging, fatigue, stress, or chronic diseases [9]. Therefore, consuming these beverages can reduce the adverse effects of poor nutrition on health and the economy [5].

Herbal infusions are well-known for their therapeutic properties. Recent research has shown advancements in the understanding of the health advantages associated with herbal infusions. The fermentation process of Kombucha resulted in an elevation in phenolic content, antioxidant activity, and a-amylase inhibition in five different types of teas [10]. The decoction of Lemongrass (*Melissa officinalis* L.) exhibited significantly greater levels of phenolic components, including rosmarinic acid, caffeic acid, and ferulic acid, with respective increases of 1.3, 1.9, and 4.6 times. Based on the citations provided in [11,12], it was observed that there was an enhancement in the DPPH radical antioxidant activity. In a separate study, it was observed that the process of kombucha fermentation resulted in an enhancement of the taste of tea and the generation of many advantageous chemical compounds [13]. Fermented beverages are considered to provide health benefits because of the presence of restructured polyphenols, gluconic, glucuronic, and lactic acids, vitamins, amino acids, and minerals resulting from the fermentation process [11].

According to recent market research, Kombucha has emerged as the most rapidly expanding fermented beverage within the global functional drink industry [14]. The conventional constituents used in the manufacture of Kombucha are black tea and sucrose. In recent times, many classifications of tea, such as white, green, oolong, and Pu-erh tea, have been used in the process of Kombucha fermentation [15]. Furthermore, a number of scholars have used other sources of raw materials, including fruits [16], vegetables, herb infusions [17], and other substances [18], to enhance the range of tastes and biological properties of kombucha. This is performed with the aim of catering to the varied preferences of customers.

In Greece and the rest of the Mediterranean countries, the consumer public is familiar with the herbs provided by nature, and an increasing part of the population is incorporating them into their daily diet. One of the most popular is mountain tea (*Sideritis* spp.), with its well-known beneficial properties. Research suggests that *Sideritis scardica* has potential pharmacological properties similar to green tea (*Camellia sinensis*) [19]. So far, it has been reported that *Sideritis scardica* has antioxidant [20,21], anti-inflammatory [22], and antibacterial effects [21]. Additionally, evidence suggests its potential to improve symptoms of Alzheimer’s disease [23]. The numerous biological properties and the vast acceptance of mountain tea in Greece as a decoction make it a very interesting raw material for producing a functional beverage. In addition, mountain tea is more interesting when combined with honey as a sweetener instead of sugar.

Honey is a highly complex natural liquid containing at least 181 substances [24]. Today, honey is one of the most nutritious food supplements and medicine, classifying it as functional food [25]. The geomorphology of Greece, with its flora and the prevailing climatic conditions, form an excellent combination for producing many different kinds of honey with particular physicochemical and organoleptic characteristics. A particularly aromatic honey produced mainly in the island regions of the country is thyme honey, known since antiquity for its bright color and intense floral aroma.

In this study, a new version of kombucha is produced with Greek raw materials. The tea (*Camellia sinensis*) is replaced with Olympus Mountain tea (*Sideritis scardica*), and the sugar is replaced with thyme honey (TH). This work aims to evaluate the effect of kombucha fermentation on the physicochemical and functional properties of this new version of kombucha with Olympus Mountain tea with honey (OMTWH).

## 2. Materials and Methods

### 2.1. Materials

Olympus Mountain tea (OMT) (2021 Bio Sideritis scardica-stems with leaves and flowers) is a certified organic agricultural product of the 2021 harvest, from an agricultural holding in Litochoro, prefecture of Pieria, was purchased from the Evripidou store evripidou.gr, Athens, Greece). Thyme honey (TH) (Lemnian land 2021) was kindly offered by Beekeeping Cooperative of Limnos, Organic Kombucha culture was obtained by Kombucha organic company (Cumbria, UK) with verification UKAS Lab tested (lab verified) and used in multiple fermentations of OMT sweetened with TH. The new Symbiotic Culture of Bacteria and Yeast (SCOBY) was placed in a SCOBY hotel and fermented only OMTWH for approximately a year. Therefore, the SCOBY used in this study originated from that SCOBY hotel. Finally, the water used was filtered tap water (AQUA-PURE filter, 3M Hellas MEPE, Attica, GR). All chemicals and enzymes were purchased from Sigma–Aldrich (St. Louis, MO, USA).

### 2.2. Kombucha Production—Fermentation Conditions

**Figure 1:**
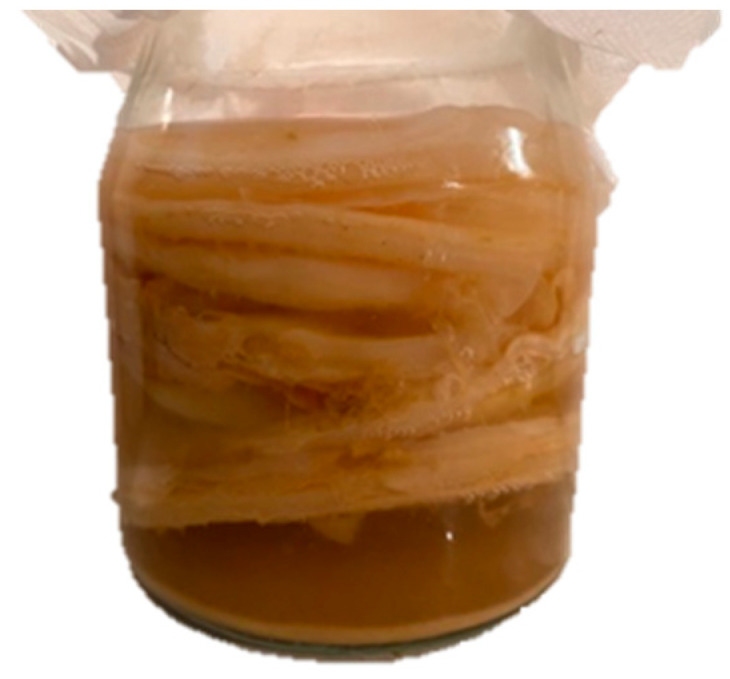
SCOBY hotel: Symbiotic Culture of Bacteria and Yeast (SCOBY) fermented Olympus Mountain Tea with Thyme Honey (OMTWH) for a period of one year.

An amount of 3.0 L of filtered water, and 5.0 g/L of mountain tea, were added and boiled for 10 min. The solution was filtered into a glass container and left to stand until it reached room temperature between 20 and 25 °C. Honey was added, 75 g/L, and stirred until dissolved. Finally, SCOBY (250.0 g) was placed into the broth. The container was covered with a linen cloth to avoid contamination and infestation, mainly by the insect Drosophila melanogaster (vinegar fly). The samples were placed in a shady place, unmoving for 4 days. On day 4, fermentation monitoring employing a taste test was started. The incubation period was terminated when optimal consuming acidity of 3.5–4.5 g/L was achieved [26]. Then the SCOBY (parent and daughter cultures) were removed and stored in glass containers (SCOBY hotel) (Figure 1). The solution was then filtered, filling the glass bottles, that were hermetically sealed.

### 2.3. Sampling

A sampling of the fermentation broth was performed daily. The pH value and titratable acidity were determined daily. The number of yeasts and acetic acid bacteria (AAB) was measured just after inoculation (day 0), day 2, and at the end of the fermentation. Analysis of color, total phenolic, and flavonoid content, a-amylase, and a-glucosidase inhibition, anticholinesterase, and antioxidant activities were performed in the broth before inoculation and at the end of the fermentation. The Kombucha liquid phase was centrifuged at 5000× *g* for 10 min, and the resultant supernatant was analyzed immediately or stored at −40 °C until further analysis.

### 2.4. Color Measurement

The color of the kombucha was measured using a spectrophotometer CM-3500d (Konica Minolta Investment Ltd., Shanghai, China). The chromatic parameters L* (lightness), a* (redness), and b* (yellowness) values were recorded. The total color difference is defined by the expression:

$${\Delta E}^{*}=\sqrt{{\Delta L}^{*2}+{\Delta a}^{*2}+{\Delta b}^{*2}}$$

Depending on the value of ΔE, the color difference between tea broth before inoculation and fermentation samples can be estimated as not noticeable (0–0.5), lightly noticeable (0.5–1.5), noticeable (1.5–3.0), well-visible (3.0–6.0), and great (>6.0) [27].

### 2.5. Determination of pH and Titratable Acidity

The pH values were determined by using an electronic pH meter Consort C931 (Turnhout, Belgium) that was calibrated at pH 4.0 and 7.0. The determination of titratable acidity was conducted using the methodology outlined in a previous study [28]. In a concise manner, subsequent to the elimination of carbon dioxide from the fermentation solution, a 20 mL portion was extracted and subjected to titration using a 0.1 molar solution of sodium hydroxide. The measurement of titratable acidity was quantified in grams per liter (g/L) of acetic acid for every individual sample.

### 2.6. Enumeration of Yeasts and Acetic Acid Bacteria

The populations of yeasts and acetic acid bacteria (AAB) in the fermentation broth were assessed using a plate pour technique. The medium used to enumerate yeasts was Dichloran Rose Bengal Chloramphenicol (DRBC) Agar (ISO) (Lab M, Heywood, UK) as described in ISO 21527-1:2008. The medium for determining the total count of AAB was ABS, a novel selective medium (AAB-Selective agar) with adding 5.0 mg/L of cycloheximide to inhibit the growth of yeasts, as previously described [29]. The tests were conducted in triplicate, maintaining consistent circumstances, with each amount being measured three times. The numbers acquired for further analysis are the average readings, which are reported as the mean ± standard deviation. The evaluation was carried out only in the broth since only the broth is typically consumed and not the pellicle. Thus, assessing the broth’s microbial composition can be deemed relatively more important than the pellicle. The populations of both AAB and yeasts were expressed as colony-forming units per mL (CFU/mL).

### 2.7. Determination of Ethanol, Sugars, Organic Acid, and Minerals

The ethanol concentration was determined using an enzymatic kit (catalog no. K-ETOH, Megazyme, International Ireland Ltd., Wicklow, Ireland). The kit’s validation was conducted exclusively inside a single laboratory setting, specifically for the analysis of Kombucha fermented beverages, fruit juices, and low-alcohol beer samples. The Ethanol Assay Kit, which is readily accessible for commercial purchase, includes all necessary components for conducting the examination. Quantification is reliant on the process of ethanol oxidation to acetaldehyde by the action of alcohol dehydrogenase, followed by the further oxidation of acetaldehyde by acetaldehyde dehydrogenase, resulting in the conversion of NAD+ to NADH. The determination of sugars, acids, and minerals was conducted using the Miura one, an enzymatic analyzer manufactured by TDI (Barcelona, Spain), in accordance with the instructions provided by the provider.

### 2.8. Vitamin C

Vitamin C was evaluated with a Spectrophotometric method [30]. An amount of 1.0 mL of OMTWH before inoculation and after the fermentation in 3% metaphosphoric acid was sampled and combined with 9.0 mL oxalic acid in EDTA, 2.0 mL of 50%(*v*/*v*) aqueous solution of sulfuric acid in water, 4.0 mL of 5% (*w*/*v*) ammonium molybdate aqueous solution and mixed thoroughly. Absorbance was measured at 705 nm at room temperature for 3 min on a Spectrophotometer Rayleigh VIS-7220G (Rayleigh Analytical Instruments, Beijing, China). A standard curve of known vitamin C concentrations was used to quantify the vitamin C in samples under investigation.

### 2.9. Vitamins of the B-Complex

Vitamins of the B-complex were identified and quantified by HPLC, using a Shimadzu 2030C prominence-i system with PDA detector and a Phenomenex Luna C18(2) analytical column (4.6 mm × 250 mm, particle size 5.0 μm) as previously described [31]. Briefly, 0.025% (*v*/*v*) trifluoroacetic acid in water and acetonitrile as mobile phases A and B were used in a gradient elution system with 2% B (98%A) at 0 to 5 min, 25%B (75%A) at 5 to 11 min, 45% B (55% A) at 11–15 min, 40% B (60%A) at 20 min, and 2%B (98%A) at 22 min. Injection volume and flow rate were 20 μL and 1.0 mL/min, respectively. A signal was recorded from 190 to 800 nm, and chromatographs were registered at 198 nm. Identification and quantification were performed with vitamin B-complex analytical standards.

### 2.10. Total Phenolic Content

The Folin–Ciocalteu method was used to determine the total phenolics content [32]. First, the reaction mixture was prepared by mixing 20.0 or 30.0 μL of the samples, 780.0 or 770.0 μL of distilled water, 50.0 μL of Folin–Ciocalteu reagent, and 150.0 μL of sodium carbonate (20.0%, *w*/*v*). After incubation at 40 °C for 30 min, absorbance was measured at 765 nm. Subsequently, the void was produced by the substitution of the specimen with distilled water. The calibration standard used in the study was Gallic acid, and the outcomes were quantified in terms of Gallic acid equivalents, specifically in micrograms per milliliter of the sample (μg GAE/mL).

### 2.11. Total Flavonoid Content

The quantification of total flavonoid content was conducted by the use of a colorimetric test [33]. First, ethanol was mixed with 300.0 μL of either OMTWH broth before inoculation or after fermentation to bring the final volume to 740.0 μL. After that 30.0 μL NaNO_2_ (5% *w*/*v*) were added, and the mixture was stirred. Following a time interval of 5 min, a volume of 30.0 μL of AlCl_3_ solution with a concentration of 10% *w*/*v* was introduced into the system and then left undisturbed for a duration of 6.0 min. Next, 200.0 μL 1.0 M NaOH were added to the mixture followed by vigorous stirring. Finally, the blank was prepared by replacing the sample with distilled water. The absorbance at a wavelength of 510 nm was measured immediately after the mixing process. Rutin was used as a calibration standard, and results were expressed as rutin equivalents (RE) in μg per mL of the sample (μg RE/mL).

### 2.12. Analysis of Antioxidant Activity

The Antioxidant capacity of Kombucha beverages was analyzed using 1,1-diphenyl-2-picrylhydrazyl (DPPH) and 2,2′-azinobis-3-ethylbenzthiazoline-6-sulfonic acid (ABTS) assays. The DPPH assay was conducted according to the procedure previously reported [34]. The analytical protocol included the mixing of 30.0 to 40.0 μL of each sample with 1.0 mL of a DPPH solution (0.6 mM in methanol). Following a 15 min incubation period in a light-restricted environment at ambient temperature, the reduction in absorbance at a wavelength of 515 nm was quantified. The ABTS assay was carried out following the procedure as previously described [35]. Amounts of 20.0 to 30.0 μL of each sample were mixed with 1.0 mL of ABTS working solution. After the mixtures were kept for 15 min in the dark at room temperature, the decrease in absorbance at 734 nm was measured. The control group was comprised of samples that did not include any tea solutions. The IC_50_ values were determined as the concentration of an antioxidant necessary to decrease the starting concentrations of DPPH or ABTS by 50%. The inhibition activity of DPPH^●^ or ABTS^●+^ was determined using the following equation: Inhibition activity % = [(A_Control_ −A_Sample_)/A_Control_] × 100. IC_50_ was calculated from the graph, plotting the inhibition percentage against μL of sample.

### 2.13. α-Amylase and α-Glucosidase Inhibition Assays

#### 2.13.1. α-Glycosidase Inhibition Assay

The evaluation of α-glycosidase inhibition by OMTWH broths before inoculation and after the fermentation was measured using a methodology described elsewhere [34] with modifications. Briefly, 15.0 to 20.0 μL of samples and 0.1 M phosphate buffer (pH 6.9, to the volume of 700.0 μL) containing 10.0 μL of α-glucosidase solution (33.3 U/mL) were incubated at 37 °C for 10 min. Then, 50.0 μL of 5.0 mM p-nitrophenyl-α-D-glucopyranoside solution in 0.1 M phosphate buffer (pH 6.9) was added to each sample. Finally, the reaction mixtures were incubated at 25 °C for 5.0 min before reading the absorbance at 405 nm in the spectrometer (SPECTROstar Nano, BMG Labtech, Ortenberg, Germany). The α-glycosidase inhibitory activity was expressed as percentage inhibition and calculated as follows: Inhibition (%) = [(A_Reference_ − A_Sample_)/A_Reference_] × 100. The IC_50_ for each sample was estimated using the fitted straight line (linear regression) plotted with the data derived (% Inhibition values) against μL of sample.

#### 2.13.2. α-Amylase Inhibition Assay

Inhibition of α-amylase was studied using the Caraway–Somogyi iodine/potassium iodide (IKI) method, as previously [36], with some modifications. The sample solution (200.0 to 250.0 μL) was mixed with α-amylase solution (1000.0 μL, 0.01% *w*/*v*) in phosphate buffer (pH = 6.9 with 6.0 mM sodium chloride) and incubated for 10.0 min at 37 °C. After preincubation, the reaction was initiated by adding starch solution (500.0 μL, 0.5% *w*/*v*). In a similar manner, a void was created by including the sample solution in all reaction reagents in the absence of the enzyme (α-amylase) solution. The reaction mixture was incubated for 10.0 min at 37 °C. The reaction was stopped by adding HCl (250.0 μL, 1.0 M) and after adding an iodine-potassium iodide solution (1000.0 μL). The absorbances of the sample and blank were measured using a Lambda 25 spectrophotometer (Perkin Elmer, Norwalk, CT, USA) at a wavelength of 630 nm. The α-amylase inhibitory activity was expressed as percentage inhibition and calculated as follows: Inhibition (%) = [(A_Reference_ − A_Sample_)/A_Reference_] × 100. The IC_50_ for each sample was estimated using the fitted straight line (linear regression) plotted with the data derived (% Inhibition values) against μL of sample.

### 2.14. Anticholinesterase Assays: AChe and BChe Inhibition

The Acetylcholinesterase (AChE) and Butyrylcholinesterase (BChE) activities were determined using a spectrophotometric method [37] with some modifications. Briefly, 1500.0 μL of 0.1 mM sodium phosphate buffer (pH = 8.0), 50.0 μL of 5,5′-dithio-bis-(2-nitrobenzoic acid) (DTNB), 150.0 to 200.0 μL of the sample, and 25.0 μL of enzyme solution were mixed and incubated for 15.0 min at 25 °C. After that, 10.0 μL of acetylthiocholine iodide (ACTHI) or butyrylthiocholine iodide (BCTHI) was added. Then the final blend was incubated for 25.0 min at 25 °C, and the absorbance was measured at 412 nm (Lambda 25, Perkin Elmer, Norwalk, CT, USA). The blank was measured without extract. The enzyme activity inhibition percentage was calculated as: % inhibition = 100 × (A_Blank_ − A_Sample_)/A_Blank_. Results were expressed as the mean ± standard deviation of three replicate values concerning the IC_50_ values for AChE and BChE activities.

### 2.15. Statistical Analysis

The findings were reported in terms of the mean value plus or minus the standard deviation (SD) based on three repeated measurements. The statistical significance of the means was assessed using a one-way analysis of variance (ANOVA). Independent sample Test, and Mann–Whitney Test using SPSS. Pearson and Spearman’s correlation coefficients evaluated correlations. The confidence limits were set at *p* < 0.05.

## 3. Results and Discussion

### 3.1. Macroscopic Changes during Fermentation. Mat Formation, Color Changes, and Clarity of the Beverages

In all solutions, an off-white cellulose membrane was formed on the first to the second day of fermentation, occupying the entire surface with a thickness that increased with the passage of days. The primary characteristic of kombucha beverages visible to the naked eye is the formation of the cellulose pellicle layer that floats on the product’s surface [38]. A change in color towards lighter and turbidity towards clearer was also observed in the solutions with time, which was noted by other investigators [39], where it was reported to be a result of the breakdown of polyphenols into smaller molecules due to enzymatic action by bacteria and yeasts in the acidic environment of kombucha. Also, the beverage started to smell fermented, and gas bubbles appeared from the carbonic acid produced during the fermentation. Finally, the mother culture sank to the bottom of the tea broth, which remained under the newly formed daughter culture, as previously described [38]. The production of this mat occurs as a result of the development of a delicate cellulose layer that serves as a substrate for the attachment of bacterial and yeast cell masses [38]. Cellulose is synthesized by the bacterial component of the microbial consortium. The primary agent involved in the formation of cellulose is Acetobacter xylinum. The presence of this cellulose network facilitates the interaction between the bacteria and the yeasts [40].

The results of the chromatic parameter changes confirmed the visual observation and are presented in Table 1. The total color differences (ΔE*) of OMTWH samples before and after fermentation were higher than 10, which showed a “great” magnitude of the color difference between OMTWH broth before inoculation and after fermentation in all samples.

### 3.2. pH and TA

**Figure 2:**
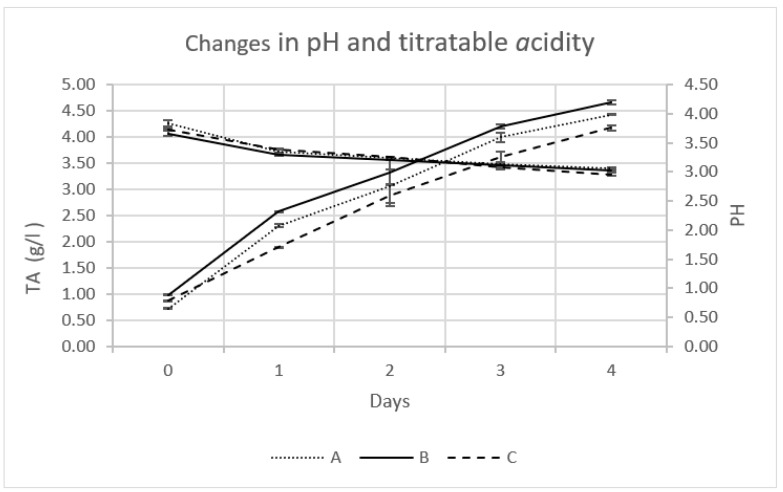
Changes in the pH values and titratable acidity at the time of inoculation (day 0) and during fermentation (days 0 through 4) of OMTWH. Each point is the mean ± standard deviation of three independent measurements for samples A, B and C.

The average changes in pH and titratable acidity during the fermentation of OMTWH are presented in Figure 2. Each average value was obtained from three observations. After fermentation, a statistically significant decrease in the pH (*p* < 0.05) was observed. During fermentation, the pH values decreased almost the same for all three samples (A, B, C).

The pH value of the OMTWH was approximately 5.90 ± 0.07, dropping to about 3.83 ± 0.05 for sample A, 3.66 ± 0.04 for B, and 3.73 ± 0.02 for C, immediately after the inoculation. The small differences in the inoculation cultures can explain the slight differences. On the first day, there was a reduction in the pH value ranging from 0.35 to 0.47 units. Subsequently, on the subsequent days, the drop in pH was constrained to a range of 0.07 to 0.18 units each day. At the end of the process, on the fourth day, the pH value reached 3.06 ± 0.02 for sample A, 3.02 ± 0.01 for B, and 2.96 ± 0.03 for C. These values are within the range considered safe for human consumption, which ranges from 2.5 to 4.2 [41]. Values below pH 2.5 have a high concentration of acetic acid, posing a risk to consumers’ health. Likewise, pH values > 4.2 may compromise the beverage’s microbiological safety.

After fermentation, a statistically significant increase in the titratable acidity (TA) (*p* < 0.05) was observed. TA increased from the beginning until the end of the fermentation process similarly for all samples (Figure 2). After the inoculation, samples A, B, and C had TA values expressed in acetic acid (g/L) of 0.73 ± 0.01, 0.99 ± 0.01, and 0.88 ± 0.01, respectively. During the fermentation, TA values increased more linearly than PH’s and, on the fourth day, reached 4.45 ± 0.02, 4.69 ± 0.04, and 4.24 ± 0.05 for samples A, B, and C.

The decrease in pH values (Figure 2) follows the increase in acidity due to the Kombucha fermentation. These changes result from the metabolic activity of yeasts and AAB that produce mainly acetic acid. As it is evident, changes in titratable acidity towards the end of the fermentation are more significant than changes in pH, which the buffer capacity of the fermented broth could explain. Specifically, the process of fermentation results in the emission of carbon dioxide. Subsequently, the resulting aqueous solution of carbon dioxide dissociates, giving rise to the formation of the amphiprotic hydrocarbonate anion (HCO_3_^−^). This anion can react quickly with H^+^ released from organic acids. This can cause more organic acid production but a smaller decrease in pH values, thus contributing to the buffer character of the system [26]. Several other researchers have also noted similar pattern of pH and TA fluctuations [42,43], which is often seen throughout the fermentation process of Kombucha. Based on the aforementioned information, the total acidity (TA) was used as a pivotal parameter in identifying the completion of kombucha fermentation, rather than relying on pH levels. In order to obtain a beverage with a desirable sourness, it is recommended to conclude the fermentation process when the total acidity (TA) reaches the optimal range of 4–4.5 g/L. This recommendation is supported by the experiences of long-term users of kombucha drinks [26]. In our case, optimal consuming acidity was obtained in less than four days. In another study, the same acidity was received after nine days of fermentation of black tea and sugar [44].

The drop in pH values and the increase in total acidity are fundamental indications that the fermentation of OMTWH from a symbiotic kombucha culture is progressing. Other parameters that suggest an active process of fermentation in OMTWH are mat formation, observed towards the end of the first day of fermentation, the color changes confirmed by the chromatic parameter changes before and after the fermentation, and residual sugars.

Immediately after inoculation, there was a correlation between pH with TA in the samples. Sample B, with the highest total acidity, had the lowest pH value, and A, with the lowest total acidity, had the highest pH value. However, during fermentation, this linearity ceased to exist. The above is because we measure the acids without considering their strength by measuring total acidity. In contrast, with pH, we estimate the acidity by considering the power of each acid involved in forming its acidic taste.

### 3.3. Yeast and Acetic Acid Bacteria Count

**Figure 3:**
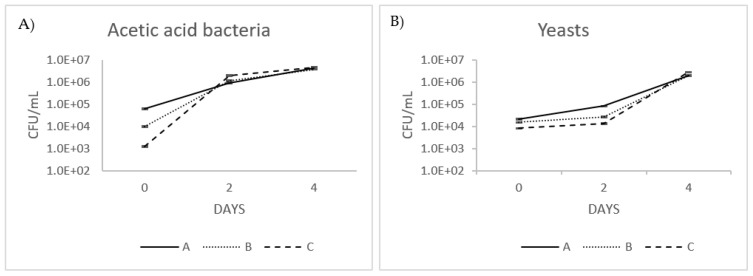
(**A**) Acetic acid bacteria count at days 0, 2, and 4. Each point is the mean ± standard deviation of three independent measurements, for samples A, B, and C. (**B**) Yeasts count at days 0, 2, and 4. Each point is the mean ± standard deviation of three independent measurements for samples A, B, and C.

Figure 3A,B show the average changes in microbiological parameters observed throughout the process of fermentation. Following the inoculation stage, significant variations in the quantities of yeasts and acetic acid bacteria (AAB) were observed across the samples. This discrepancy may be attributed to the use of three distinct symbiotic cultures of bacteria and yeast (SCOBYs), each potentially containing varying quantities of viable cells. Nevertheless, by the end of the fermentation, acetic bacteria and yeasts were uniform among the three samples.

In more detail, just after inoculation, the broth’s initial number of acetic bacteria was at the level of 6.2 × 10^4^ ± 5.4 × 10^3^, 1.01 × 10^4^ ± 7.4 × 10^2^, and 1.2 × 10^3^ ± 1.3 × 10^2^ CFU/mL for samples A, B, and C, respectively. The total count of bacteria increased quickly daily to the maximum of 4.3 × 10^6^ ± 2.1 × 10^5^, 3.9 × 10^6^ ± 1.4 × 10^5^, and 4.7 × 10^6^ ± 2.7 × 10^5^ CFU/mL on fermentation’s 4th and final day.

The initial number of yeasts was at 2.1 × 10^4^ ± 1.7 × 10^3^, 1.5 × 10^4^ ± 9.6 × 10^2^, and 8 × 10^3^ ± 1.7 × 10^2^ CFU/mL for samples A, B, and C, respectively. The total count of yeasts increased slowly until day 2 of fermentation and then rapidly reached the peak of 2 × 10^6^ ± 1.0 × 10^5^, 2.0 × 10^6^ ± 7.4 × 10^4^, and 2.9 × 10^6^ ± 4.9 × 10^4^ CFU/mL.

The increase was indicative of the ability of the OMTWH to act as a suitable substrate to sustain the proliferation of the microorganisms of the Kombucha symbiotic culture.

The total count of yeasts and AAB by the end of fermentation is comparable with previous investigations. Bacterial cell and yeast cell numbers are generally thought to reach 10^4^–10^6^ CFU/mL in a kombucha culture that has been allowed to ferment for approximately ten days [45]. Another study showed counts of mesophilic bacteria (6.93 × 10^6^ CFU/mL) and yeasts (7.52 × 10^5^ CFU/mL) after fermentation at 28 °C for 10 days, close to those obtained in our study [46]. Similarly, Sreeramulu and others [43] obtained 4.48 log CFU/mL yeast cells and 5.3 logs CFU/mL bacterial cells in fermentation liquid after six days of the process. Also, Teoh and coauthors [45] found the count of individual yeast species on the sixth day of the process between 5 and 7 log CFU/mL. Some differences in chemical parameters, process duration, and cell counts may be expected because kombucha does not have a standardized microbiological and chemical composition [45]. In another study [47], during the kombucha fermentation of black tea sweetened with sugar, the viable counts of yeasts increased and followed the same trend as those of AAB. Also, in contrast with our study, cell concentrations of yeasts were generally higher than those of AAB, and the growth of yeasts was faster than that of the bacteria. The above concurs with the findings of [48,49].

The above can be explained as follows: Yeast and bacteria in the Kombucha symbiotic culture use substrates in different and complementary ways. At the initial stage of fermentation, yeasts hydrolyzed sucrose to glucose and fructose, which were further utilized, with a preference for fructose, to produce ethanol and carbon dioxide [40,50]. Then AAB used glucose to produce gluconic acid and ethanol to produce acetic acid [38]. The metabolic activity of AAB has been reported to be lower than that of yeasts because the bacteria’s nutrient source must initially be utilized and produced by yeasts [51]. In our study, this is not the case, as the growth of acetic bacteria was faster than that of yeasts. The explanation lies in the chemical composition of the sugars in honey. Honey is composed mainly of fructose and glucose, while sugar is composed of sucrose. During the fermentation process, sucrose is first broken down into glucose and fructose by the yeasts through the enzyme invertase. In honey, the sugars are readily available, so the AAB can start their action without the prior action of the yeasts, mainly using the readily available glucose to produce gluconic acid and cellulose for the mat formation.

Similarly, the yeasts hydrolyze some small amounts of sucrose that may be present in the honey and, at the same time, produce alcohol from glucose, preferably fructose, which is then used by the AAB for the production of acetic acid. The growth rate of the yeasts is lower than that of the acetic bacteria during the first two days, and then there is a reversal of this trend until the end of fermentation. AAB have the upper hand in the conditions prevailing at the beginning of fermentation, while yeasts have the upper hand after the second day. The optimum temperature range for yeast fermentation is between 30 and 35 °C [52], and the optimal temperature for the growth of AAB is 25 to 30 °C [53]. So, the temperature of 25 °C at which the fermentation took place was more favorable for the growth of AAB. The growth rate of AAB, as aerobic microorganisms, is negatively affected after day 2, when the cellulose membrane has formed on the vessel’s surface, converting the fermentation conditions to anaerobic. Yeasts, respectively, as facultative anaerobes, are favored. Liu and colleagues studied the relationship of the Kombucha organisms [54]. They found that the yeasts’ ethanol production assisted the bacterial production of acetic acid, and acetic acid production may further stimulate yeast production of ethanol. Finally, it was noted that the simultaneous production of ethanol and acetic acid prevents the competition of other microorganisms. This relationship illustrates the defined level of symbiosis and compatibility between the organisms in the tea colony.

After storing the OMTWH kombucha beverage under refrigeration at 4 °C for ten days, the number of AAB and yeasts mildly decreased. Decreases were found statistically significant (one-way ANOVA). The number of AAB decreased from 4.3 × 10^6^ ± 2.7 × 10^5^ to 2.8 × 10^6^ ± 1.7 × 10^5^ CFU/mL (*p* < 0.05) for sample A, 3.8 × 10^6^ ± 1.1 × 10^5^ to 1.9 × 10^6^ ± 0.9 × 10^5^ CFU/mL (*p* < 0.05) for B, and 4.7 × 10^6^ ± 2.2 × 10^5^ to 2.9 × 10^6^ ± 1.2 × 10^5^ CFU/mL (*p* < 0.05) for C. Likewise, the number of yeasts decreased from 2 × 10^6^ ± 0.9 × 10^5^ to 1.4 × 10^6^ ± 0.9 × 10^5^ CFU/mL (*p* < 0.05) for sample A, 1.9 × 10^6^ ± 1.1 × 10^5^ to 1.2 × 10^6^ ± 1.0 × 10^5^ CFU/mL (*p* < 0.05) for B, and 2.9 × 10^6^ ± 1.3 × 10^5^ to 2.1 × 10^6^ ± 0.9 × 10^5^ CFU/mL (*p* < 0.05) for C. This is in accordance with a previous study [55], where the survival rate of yeast and AAB on the 10th day of keeping kombucha beverages refrigerated at 4 °C was 73.97% and 54.09%, respectively.

The results show that mountain tea with honey is a suitable substrate, respectively, with black tea and sugar, for microbial growth in symbiotic kombucha culture fermentation.

### 3.4. Changes in Sugars, Ethanol, Organic Acids, Minerals

#### 3.4.1. Sugars

Sugar in the fermentation medium is used as a carbon source for the growth of microorganism cells, in addition to the metabolic processes that produce cellulose and metabolites in the form of specific organic acids [38].

On day zero, before inoculation, the total sugar content was 57.0 ± 2.8 g/L, corresponding to the honey added (75.0 g/L). Glucose and fructose contents were 23.7 ± 0.8 g/L and 26.7 ± 1.3 g/L, respectively. The results are in agreement with the literature. It has been reported that about 83% of honey is composed mainly of sugars and that 22 different sugars have been identified and classified into monosaccharides, disaccharides, and oligosaccharides [56]. The monosaccharides fructose (32–44%) and glucose (23–38%) are the main honey sugars [57]. In almost all honey types, fructose is the primary sugar. Nevertheless, some types of honey, such as those derived from Brassica napus (rape), Taraxacum officinale (dandelion), and Trichostema lanceolatum (blue curls), have higher levels of glucose concentration [58]. These particular honeys deviate from the established norm. More than 45 di-, tri-, and other oligo- and polysaccharides have been detected in honey in small quantities (5–15%) [57,59,60,61]. Maltose (7%) and sucrose (1%) are essential honey disaccharides [62].

After fermentation, total sugars decreased by 21.0% for sample C and 26.3% for samples A and B. The drop in fructose value was almost double that of glucose for samples A and B. At the same time, it was similar for sample C. Thus, the fructose value after fermentation dropped to 17.1 ± 0.76 and 18.6 ± 0.99 g/L for A, B, and 21.0 ± 0,91 g/L for C. Glucose dropped to 20.4 ± 0.7, 20.1± 0.8 and 18.6 ± 0.7 g/L for samples A, B, and C, respectively. Generally, fructose content was lower than glucose during fermentation, suggesting that fructose was preferred as the carbon source by yeast cells. The results are consistent with previous studies [38,63]. Yeast and bacteria in the Kombucha consortium use substrates in different and complementary ways. Yeast cells hydrolyze sucrose to glucose and fructose and then produce ethanol, with a preference for fructose as a substrate [64]. In contrast, AAB utilize glucose to produce gluconic acid and cellulose and ethanol to produce acetic acid [39,44,65,66,67].

More analytically, in the pentose phosphate pathway, the enzyme glucokinase converts glucose to glucose-6-phosphate (G6P), and then G6P is converted to glucose-1-phosphate (G1P) with the help of phosphoglucomutase enzyme and converted back to uridine diphosphate (UDP) with pyrophosphorylase enzyme until it is converted to cellulose with the help of cellulose synthase enzymes [68]. The result of cellulose formation will be seen as a pellicle on the surface of the fermentation medium. Besides producing cellulose, the metabolic process of Acetobacter xylinum will also produce primary metabolites in the form of acetic acid and other organic acids, including gluconic acid, glucuronic acid, malic acid, tartaric acid, citric acid, butyrate, and lactic acid [38,69].

A slight differentiation is observed in sample C, with higher glucose and lower fructose consumption compared with samples A and B. From the results of the microorganism count, a higher number of acetic bacteria is recorded in sample C after the first day of fermentation and a lower number of yeasts until at least the third day compared with samples A and B. So, the higher number of acetic bacteria in C is responsible for the higher glucose consumption for conversion to gluconic acid and cellulose. In contrast, the reduced number of yeasts during the more extended fermentation period is reflected in the reduced consumption of fructose to ethanol compared with A, B.

The sugar consumption and acid production observed suggest an active fermentation process in the OMTWH.

After 30 days of refrigeration at 4 °C, a decrease in the total sugars value of 47.4%, 42.1%, and 36% is observed for samples A, B, and C, respectively. The glucose value shows a decrease of 22.8% for A and B and 25.3% for C. Finally, the fructose value decreased by 67.4%, 62.9%, and 41.6% in samples A, B, and C, respectively. We conclude that fermentation continues at a slow rate during refrigeration at 4 °C.

#### 3.4.2. Acids

On day zero, before inoculation, gluconic, acetic, citric, tartaric, and lactic acids were found at concentrations of 0.46 ± 0.01 g/L, 0.02 ± 0.00 g/L, 0.03 ± 0.00 g/L, 0.69 ± 0.02 g/L, and 0.09 ± 0.00 g/L, respectively. Those concentrations corresponded to the honey added (75.0 g/L) and the published scientific literature.

Honey contains organic acids in equilibrium with their corresponding lactones [58,70]. They contribute to honey acidity and electrical conductivity. Some honey’s organic acids will likely come directly from nectar or honeydew (citric, malic, and oxalic). However, the vast majority of them are produced from nectar and honeydew sugars by the action of enzymes secreted by bees during ripeness and storage (formic acid and others) [58,71,72]. Furthermore, in the process of storage, the osmophilic yeasts enzymatically convert honey sugars into alcohols and subsequently organic acids, facilitated by the action of glucose oxidase. This metabolic activity results in the production of various acids, including acetic acid, which might serve as potential markers of honey fermentation [73,74]. Gluconic acid is the main honey organic acid, representing 70–90% of the total acidity [75]. It comes from glucose through the action of glucose oxidase.

The presence of gluconic acid and gluconolactone in honey is known to exist in an equilibrium state [72,73]. In addition to gluconic acid, a comprehensive analysis of honey has identified over 30 non-aromatic organic acids, including acetic, butyric, citric, formic, lactic, maleic, malic, oxalic, fumaric, pyroglutamic, succinic, pyruvic, and tartaric acids, among others [74]. The presence of organic acids in honey serves as a distinguishing feature that reflects its botanical source [76,77,78].

L-tartaric acid was present in all analyzed samples ranging from 0.57 to 0.69 g/L. The results were in accordance with [79], where this acid ranged from 0.508 to 0.698 g/L.

After fermentation, the dominant acid in the beverage for samples A and B was acetic acid with a concentration of 2.91 ± 0.10 g/L, 2.85 ± 0.11 g/L, and gluconic acid for C with a concentration of 2.84 ± 0.08 g/L.

The second most abundant acid was gluconic acid, with concentrations of 1.59 ± 0.06 g/L, 1.72 ± 0.06 g/L for samples A and B, and acetic acid, with a concentration of 2.28 ± 0.07 g/L for C. All the other acids remained unchanged after fermentation. The gluconic to acetic acid ratio was 0.55 ± 0.02, 0.60 ± 0.02, and 1.25 ± 0.05 for samples A, B, and C, respectively. The most significant ratio is that of sample C, as expected by the equivalent consumption of sugars during fermentation. Also, the gluconic and acetic acid values correlate with the corresponding values of glucose and fructose, respectively. These conclusions are in agreement with the literature.

As anticipated, acetic acid is the predominant organic acid present in kombucha beverages. All examined kombucha beverages yielded good results for the presence of this particular constituent, which is the most discernible byproduct derived from the fermentation process of kombucha. The manufacturing of AAB is derived from ethanol [39]. In a study conducted by Sievers et al. [44], it was shown that after a fermentation period of eight days, two distinct Kombucha beverages derived from different tea fungus cultures exhibited acetic acid concentrations of around 3.0 and 4.0 g/L, respectively. The data were obtained using enzymatic analysis. According to the findings of an HPLC examination, it was shown that acetic acid constituted the majority of the organic acids present, representing 62% of the titratable acidity [48]. Similarly, acetic acid was the primary acid produced in kombucha with black and green tea, with a concentration close to 3.0 g/L [80]. Acetic acid was also the main organic acid found in tea fungus metabolites in a recent study [27], and the concentrations of acetic acid in Zijuan tea-based kombucha increased with prolonged fermentation time.

Additionally, gluconic acid was the other major organic acid found, and it reached a maximal concentration of 2.3 g/L in Zijuan tea-based kombucha on day 14. As reported, acetic and gluconic acids were the main organic acids in kombucha beverages [81]. They found that both the time of fermentation and the type of substrate significantly affected the concentrations of acetic acid and gluconic acid in the kombucha and reached the maximum concentration of 28.0 and 1.10 g/L, respectively. However, the presence of a high acetic acid negatively impacts the overall acceptability of kombucha due to its vinegar-like taste. On the contrary, gluconic acid was found to be beneficial with its mild, soft, and refreshing taste [82]. It is essential to highlight that a higher content of gluconic acid in kombucha is related to a better sensory quality of the beverage [83].

Sample C indicates that it is possible, with honey as a carbon substrate and a low count of yeast cells at the beginning of fermentation, that a more significant ratio of gluconic acid to acetic acid can be achieved with a positive impact on the overall acceptability of kombucha. The flavor and aroma profile of a batch of tea Kombucha is contingent upon the constituents included in the fermentation medium. The parameters included in this set of variables consist of the acetic acid to gluconic acid ratio, the carbon dioxide concentration, and the concentration of organic acids. The flavors generated by gluconic acid are characterized by their delicate nature, but those resulting from volatile acetic acid exhibit astringent and acidic qualities. Therefore, obtaining the desired quality of broth is possible by controlling the fermentation conditions.

Organic acids present in kombucha also exhibit bioactivity. They play an essential role in biological processes through their involvement in metabolic pathways as intermediate or final products [17]. A study has revealed the ability of acetic acid at a concentration of 1.0 g/L to inhibit the growth of harmful bacteria that are capable of producing spores [84]. Furthermore, the acidification of the cytoplasm and the accumulation of dissociated acid anions to potentially dangerous levels, induced by acetic acid and other organic acids, might have an impact on antibacterial efficacy [12]. The results also justify the lower total acidity expressed as acetic acid (g/L) in combination with the lower pH value in sample C compared with A and B. The gluconic to acetic acid ratio is 0.55 ± 0.02, 0.60 ± 0.02, and 1.25 ± 0.05 for samples A, B, and C, respectively. Sample C has a higher ratio of gluconic to acetic acid, which is a stronger acid (pKa = 3.86) than acetic acid (pKa = 4.76), thus further reducing the pH in sample C. By reducing the contents of the acids to total acidity expressed as acetic acid, the value of sample C is lower than that of sample A and B. The molecular weight of gluconic acid is 196,16 g/mol, while that of acetic acid is 60.05 g/mol. Finally, the decrease in total sugars correlates with the total acidity in the samples.

After 30 days of refrigeration at 4 °C, there is an increase in the value of acetic and gluconic acid in the samples, as expected from the corresponding decrease in the sugar values. Acetic acid has a concentration of 3.54 ± 0.12 g/L, 3.51 ± 0.13 g/L, 2.85 ± 0.09 g/L, and gluconic acid 1.80 ± 0.05 g/L, 1.95 ± 0.06 g/L, and 3.14 ± 0.16 g/L for samples A, B, and C, respectively. There is also a production of 0.02 ± 0.00 and 0.01± 0.00 g/L of malic acid in samples A and B. Malic acid can be produced by yeasts [85] and constitutes a byproduct of fermentation that helps detoxify the liver [86].

#### 3.4.3. Ethanol

On day zero, before inoculation, the ethanol content was zero. However, after fermentation, the ethanol content reached 5.91 ± 0.75, 5.26 ± 0.58, 3.87 ± 0.63 g/L or 0.75 ± 0.10, 0.67 ± 0.07, 0.49 ± 0.08% (*v*/*v*) for A, B, and C, respectively.

The higher number of yeasts can explain the higher values in ethanol content in samples A and B during the more extended fermentation period that consumed more fructose to produce more ethanol than C.

The values obtained were as per previous studies in traditional kombucha beverages. According to a recent study, it was shown that the concentration of ethanol in a kombucha beverage reached 6.3 g/L when the fermentation process used a sugar concentration of 50.0 g/L [87]. According to Chen and Liu, ethanol concentration in kombucha increases with fermentation time, reaching an approximate maximum value of 5.5 g/L on the 20th day of fermentation [48]. Furthermore, Cardoso and colleagues found that the green and black tea kombucha had an alcoholic content of 7.29 g/L and 4.90 g/L [80]. According to a separate investigation, the ethanol concentration reached 0.78% on the seventh day and peaked at 1.10% on the tenth day of fermentation [63].

After refrigeration for 30 days at 4 °C, ethanol content changed significantly (*p* < 0.05) in all samples and reached values of 13.41 ± 0.09, 13.10 ± 0.31, 12.84 ± 0.15 g/L or 1.70 ± 0.01, 1.66 ± 0.04, 1.61 ± 0.02% (*v*/*v*) for A, B, and C, respectively. The above trend is reflected in the total sugars decrease of 47.4%, 42.1%, and 36% for A, B, and C. This indicates an ongoing fermentation, and the insignificant change in the acetic and gluconic acid values in the samples, respectively, that can be explained by the anaerobic conditions in the sealed bottles.

The results are in line with the study of Talebi and colleagues, where the ethanol concentration in all commercial kombucha products examined was 1.12–2.00% (*v*/*v*) [87]. Moreover, it was noted that the ethanol concentration of commercial kombucha products changes with time. Longer storage times resulted in higher ethanol concentrations in both sample products at 4 °C and room temperature. Moreover, previous studies have provided evidence indicating that the addition of sugar and the time of fermentation have a crucial role in determining the concentration of alcohol [88].

#### 3.4.4. Minerals

The study of trace elements found calcium and potassium in concentrations of 31.0–33.0 ± 1.2 g/L and 64.0 ± 2.2 g/L, respectively, unchanged during fermentation, derived from honey, mountain tea, and water. The essential minerals in honey are potassium, sodium, calcium, and magnesium [89]. Potassium is the main one, standing for 80% of the total, due to its quick secretion by nectar sources [90].

### 3.5. Vitamin C

After fermentation, a statistically significant increase in the vitamin C content was observed (*p* < 0.05). Vitamin C content increased from 29.12 ± 0.15 mg/L to 36.05 ± 0.95, 33.51 ± 0.76, and 30.09 ± 0.40 mg/L for samples A, B, and C after 4 days of fermentation.

Similarly, Lonǎr and colleagues commented that fermentation induces biosynthesis of vitamin C and found the quantity to be around 1 mg per 100 mL of fermented beverage [91]. Vitamin C is the most common vitamin found in kombucha beverages (15.19 mg/L black tea kombucha and 7.88 mg/L green tea kombucha) [38,92]. Additionally, water-soluble vitamins in kombucha with 0.7% sucrose and 5.0 g/L black tea were previously quantified with a value for vitamin C equal to 15.1 mg/L [93]. Malbaša and colleagues measured the maximum content of vitamin C to be 28.98 mg/L on the 10th day in a beverage produced with a combination of AAB and S. cerevisiae isolated from native kombucha and also found that his value was slightly lower (27.86 mg/L) in the traditional product at the same stage of fermentation [92].

The values of vitamin C obtained in our study are higher than those obtained for traditional kombucha beverages after fermentation. The above can be explained by the different substrates used and their vitamin C content. Sugar in the traditional kombucha beverage does not contain vitamin C. On the other hand, the most important vitamin in honey is vitamin C [94]. Vitamin C has been especially determined in honey because of its antioxidant effect, and it is often found in almost all kinds of honey [95,96,97]. The vitamin C content of ninety kinds of honey has been previously reported [98]. The results showed that vitamin C is present in all samples. In particular, multi-floral honey presented the highest vitamin C content (5.38 mg per kg honey; *p* < 0.05). In unifloral honey, the content ranged between 2.68 mg per kg honey (citrus honey) and 3.92 mg per kg honey (chestnut honey). These values were similar to those found in citrus and eucalyptus honey, while they were lower than those found in sulla honey by [31]. Moreover, other studies highlighted thyme honey, whose amount of vitamin C is ten times higher (109–149 mg/100 g) than the other kind of honey [94,95]. Moreover, both leaves and flowers from *Sideritis scardica* have a high level of ascorbic acid [99].

In kombucha fermentation, it is assumed that vitamin C is derived from glucose and synthesized by bacteria [100]. Biosynthesis of vitamin C in kombucha throughout the fermentation process is highly associated with fermentation temperature, duration of the fermentation process, symbiosis of the SCOBY culture, source of carbon atoms, and other conditions [101].

Previous studies have described that Gluconobacter species exhibit a preference for glucose and possess the ability to oxidize glucose via two distinct routes [102,103]. The first process takes place intracellularly, wherein oxidation transpires through the pentose phosphate route. On the other hand, the subsequent process happens extracellularly and entails the production of gluconic and ketogluconic acid. Furthermore, gluconic acid serves as a metabolite of acetic acid bacteria (AAB) and has beneficial effects on human health. It acts as a precursor in the manufacture of naturally occurring vitamin C. Glucose is used as a substrate for the initiation of the reaction, specifically for the production of 2-oxo-L-gluonic acid, which serves as a precursor for vitamin C. In addition to glucose, other carbohydrates that may be considered include fructose, galactose, as well as arabinose, rhamnose, xylose, sorbose, or sucrose [104]. The precise mechanisms in the metabolic production of vitamin C in kombucha fermentation are to be determined [86].

After refrigeration for 30 days at 4 °C, vitamin C content did not change significantly (*p* ≥ 0.05). The stability in the vitamin C content can be attributed to the low temperatures and pH and the acetic acid content of the beverage. Natural mild organic acids, such as acetic acid, can maintain the stability of ascorbic acid in food [105]. Lower temperatures have been indicated as a factor to inhibit ascorbic acid degradation and thus limit the accumulation of various degradation products [106]. Low acidity is an adequate environment for storing vitamin C [107].

### 3.6. Vitamin B-Complex

The results from vitamin B-complex quantification are presented in Table 2. It can be concluded that after fermentation, a statistically significant increase in vitamins B1, B2, B6, B7, and B12 content was observed (*p* < 0.05).

Similarly, in accordance with another study, tea fungal fermentation processes increased the water-soluble vitamin content in the sweet black tea infusion by 161% for vitamin B1, 183% for vitamin B6, and 231% for vitamin B12 [93]. It has also been noted that vitamins of the B complex are synthesized during kombucha fermentation [38]. Similar results in vitamin B complex were produced in the fermentation of soybeans to tempeh and the fermentation of lentils, black gram, and rice by Rhizopus species and Klebsiella pneumonia, respectively [8,108].

Also, increased levels of thiamine, pyridoxine, niacin, and pantothenic acid have been reported to be the result of lactic acid bacteria (LAB) fermentation in yogurt, buttermilk, cheese, and other fermented products [109,110]. Moreover, the vitamin B2, B3, B6, B9, and B12 content has been shown to significantly increase during fermentation in cowpea and soybean tempeh [111].

The presence of vitamins in the Olympus Mountain tea with honey before fermentation (sample A0) can be explained by the concentration of vitamin B-complex in the honey [31,112,113,114,115,116].

According to Behrendt and colleagues, a dietary supplement combining an herbal extract of *Sideritis scardica* and selected B vitamins can alleviate stress-induced impairment of executive functioning in regard to working memory, cognitive flexibility, and controlled behavioral inhibition after an intake period of six weeks [117]. These results may be relevant for individuals who must solve cognitive tasks in the presence of conflict and/or noise, e.g., in an open-plan office or in complex, potentially hazardous traffic situations during car driving.

### 3.7. Changes in Total Phenolic and Flavonoid Compounds

**Figure 4:**
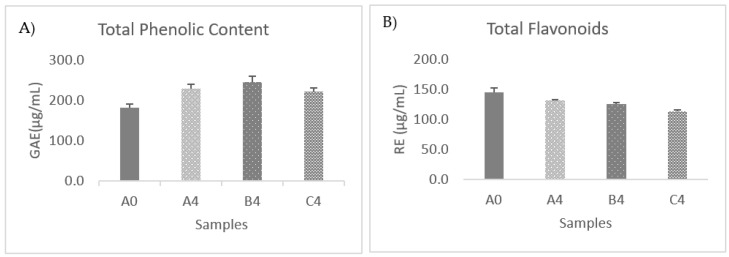
(**A**) Total phenolic content in kombucha samples before (A0) and after (A4, B4, C4) fermentation. Results are expressed as mean ± standard deviation in gallic acid equivalents (GAE) of three independent measurements. (**B**) Total flavonoid content in kombucha samples before (A0) and after (A4, B4, C4) fermentation. Results are expressed as mean ± standard deviation in rutin equivalents (RE) of three independent measurements.

After fermentation, a statistically significant increase in the total phenolic compounds (*p* < 0.05) was observed. As shown in Figure 4A, Total phenolic compounds in OMTWH increased from 182.13 ± 9.24 μgGAE/mL to 230.40 ± 10.24, 245.45 ± 15.36, and 222.99 ± 7.93 μg GAE/mL for samples A, B, and C after 4 days of fermentation. The relative amounts of total polyphenols in the OMTWH Kombucha, compared with the OMTWH, increased by 26.5%, 34%, and 22% for samples A, B, and C.

A similar trend was observed in kombucha beverages after the fermentation by other investigators [39,49,65,118,119]. The increment in the phenolic compounds was previously explained by Blanc [120]. The study showed that the enzymes (phytases, in particular) liberated by bacteria and yeast in the Kombucha consortium, and the increased acidic environment of the fermentation process, could release polyphenol compounds from the cellulosic backbone, resulting in an increase in polyphenols in the soluble fraction of the fermented beverage. It has also been identified that the enzymes liberated and low pH during the fermentation could also cause the bio-transformation or degradation of complex polyphenols to small molecules, resulting in increased phenolic compounds. This explanation has also been supported by other investigators [39,49,65,118,119].

After fermentation, a statistically significant decrease in the flavonoid content (*p* < 0.05) was observed.

As shown in Figure 4B, total flavonoid content (TFC) in OMTWH slightly decreased from 144.9 ± 7.6 μg RE/mL to 131.6 ± 1.7, 125.2 ± 2.5, and 113.5 ± 2.9 μg RE/mL for samples A, B, and C after four days of fermentation. Only minor differences in content values were found between the individual samples.

The same trend was observed in a recent study where total flavonoid content was lower in kombucha beverages with yarrow than in corresponding initial substrates and suggested that low pH in the fermented beverage caused the degradation of flavonoids [17]. In accordance with this, a decrease of 20% in the flavonoid content in Rooibos tea sweetened with sugar after kombucha fermentation has also been observed [121]. Furthermore, it has been referred that gut bacteria can cleave the C ring of flavonoids and release phenolic acid as 3-(4-hydroxyphenyl)-propionic acid and 3-hydroxyphenyl acetic acid [122]. It was, therefore, possible that yeasts and bacteria of the kombucha consortium secreted enzymes capable of catalyzing the biodegradation of flavonoids and releasing phenolic acids.

In our study, a strong positive correlation (Spearman’s correlation = −0.895, *p* < 0.01) was observed between the total phenolics compounds and the total acidity in samples A, B, and C. There is also a strong positive correlation (Spearman’s correlation = 0.939, *p* < 0.01) between the decrease in flavonoids and the corresponding decrease in pH values. It has been shown that the content of total polyphenol compounds increased linearly with the fermentation time [49]. That explains the correlation of TPC with TA, which change during fermentation more linearly than pH because of the buffer effect. On the other hand, the correlation between TF and pH is supported by a previous work, suggesting that low pH in the fermented kombucha beverage with yarrow caused the degradation of flavonoids [17].

After refrigeration at 4 °C for 30 days, total phenolics in samples A and B did not change significantly (*p* > 0.05). However, a slight increase was observed in sample C, which showed the lowest value before refrigeration. Total flavonoids decreased significantly (*p* < 0.05) by 32% in sample A, 29% in B, and 19% in C. Now, the differences in the values of flavonoids and total phenolics between the three samples were almost annihilated.

### 3.8. Antioxidant Activity Evaluation

Two different antioxidant evaluation assays, the DPPH and the ABTS scavenging abilities were applied to estimate the antioxidant capacity of OMTWH before and after Kombucha fermentation. The antioxidant properties of all samples were expressed as IC_50_ values in μL of kombucha.

**Figure 5:**
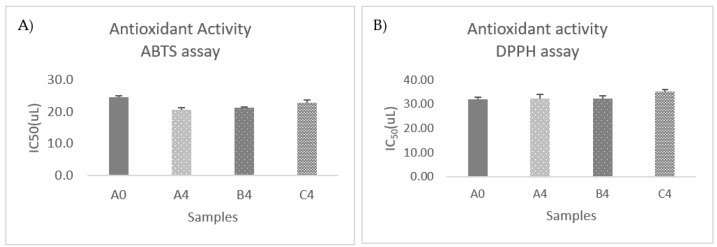
(**A**) Antioxidant Activity based on ABTS assay in kombucha samples before (A0) and after (A4, B4, C4) fermentation. Results are expressed as mean ± standard deviation in μL required for 50% scavenging of ABTS (IC_50_) of three independent measurements. (**B**) Antioxidant Activity based on DPPH assay in kombucha samples before (A0) and after (A4, B4, C4) fermentation. Results are expressed as mean ± standard deviation in μL required for 50% scavenging of DPPH (IC_50_) of three independent measurements.

The IC_50_ values for ABTS of OMTWH were estimated to be 24.7 ± 0.4 before and 20.7 ± 0.7, 21.3 ± 0.4, and 22.8 ± 0.9 after Kombucha fermentation in samples A, B, and C, respectively (Figure 5A).

The IC_50_ values of antioxidants for ABTS decreased significantly (*p* < 0.05) during fermentation, which denotes increasing antioxidant activity. On the contrary, there was no significant decrease (*p* < 0.05) in the antioxidant activity determined by the DPPH assay during fermentation (Figure 5B).

High total phenolic content enhances antioxidant activity [123]. Our study showed an increase in TPC during fermentation, explaining the enhancement of the antioxidant activity. Furthermore, it has been shown that the increase in antioxidant activity in kombucha samples mostly stems from the presence of low molecular weight polyphenols generated during the fermentation process. This observation has been made by scholars [38].

This study’s strong negative correlation between total phenolic content and ABTS IC_50_ values (Pearson Correlation = −0.837, *p* < 0.01) further confirms these points. Moreover, in a recent study, a statistically significant regression coefficient for the number of phenolic compounds and antioxidant activity determined by ABTS (r = 0.999) was observed in Kombucha tea beverages [119]. In addition, it has been demonstrated that the fermentation process with the kombucha consortium might be essential in improving soy whey beverages’ free radical scavenging ability [18].

On the other hand, DPPH IC_50_ values had a medium negative correlation with total flavonoid content (Pearson correlation = −0.629, *p* < 0.05) and no correlation with total phenolic compounds (*p* ≥ 0.05). Moreover, ABTS IC_50_ values showed no correlation with total flavonoid content (*p* ≥ 0.05). This is in accordance with the study of Gamboa-Gomez and colleagues who similarly did not find any statistically significant correlation (R = 0.3162) (*p* < 0.05) between the phenolic content and DPPH values in Eucalyptus camaldulensis and Litsea glaucescens infusions fermented with kombucha consortium [124].

The same trend was observed in a recent study, where despite the higher amount of phenolic compounds, the antioxidant activity was lower compared with the non-fermented blueberry beverage in relation to DPPH [125]. The lower antioxidant activity of the fermented beverage may be due to the changes in the anthocyanins during fermentation caused by the reduction in the medium’s pH. A previous study has found that with increasing pH, there is increased antioxidant activity, in which the constants of total deactivation of singlet oxygen by the flavonoids 3,7-dihydroxyflavone (DHF), 7-hydhydroxy flavones) and 2′,4′-dihydrochalcone (DHC) increased 5, 6, and 25 times, respectively, in the basic medium compared with the neutral medium. These constants were about 20% lower in an acid medium than in a neutral medium for HF and DHC and 6% lower for DHF [126]. The same pattern has been found when studying jambolão [127]. Thus, the lower pH of the fermented beverage may have influenced the ability to deactivate the free radicals of anthocyanins, leading to a lower antioxidant activity than the non-fermented beverage.

Jayabalan and colleagues reported that the antioxidant capacity of kombucha had been significantly affected by the composition of SCOBY, fermentation period, temperature, and other metabolites produced during fermentation [118]. Moreover, properties of antioxidants such as polarity, ionization state, and steric hindrance also seemed to contribute to their antioxidant abilities, as mentioned by other studies [28,128,129,130].

The ABTS method has extra flexibility because it can be used at different pH levels, unlike DPPH, which is sensitive to acidic pH [131,132]. When researching the impact that pH has on the antioxidant activity of a variety of substances, this information might be helpful. It is also helpful for measuring the antioxidant activity of samples extracted in acidic solvents. On the other hand, it has been mentioned that using DPPH to measure antioxidant capacity (AOC) has many drawbacks. The essay is not a competitive reaction because DPPH is a radical probe and oxidant. The loss of color in DPPH may occur by radical processes, such as hydrogen atom transfer (HAT), or reduction events, such as single electron transfer (SET). Additionally, unrelated reactions can also contribute to the loss of color. The accessibility of steric factors plays a crucial role in determining the outcome of these reactions. Thus, this test shows that small molecules with better access to the radical site have higher apparent AOC. DPPH has a relatively small linear reaction range of only 2–3-fold. Because of steric inaccessibility, the reactivity of certain antioxidants toward peroxyl radicals may be hampered or rendered inactive when it comes to DPPH. DPPH is also decolorized by reducing agents and proton transfer, contributing to inaccurate AOC interpretations. Thus, AOC is not fairly rated by the ability of antioxidants to react with DPPH [133].

Furthermore, Foti and colleagues indicated that the reaction between phenols and DPPH behaves like an electron transfer (ET) reaction. The research findings indicate that the reaction’s rate-determining stage involves a rapid electron transfer mechanism from the phenoxide anions to DPPH. The process of hydrogen atom abstraction from the neutral ArOH by DPPH becomes less favorable due to its sluggish kinetics in solvents with strong hydrogen-bond-accepting properties, such as methanol and ethanol. Furthermore, the researcher discovered that the presence of adventitious acids or bases in the solvent may significantly impact the ionization equilibrium of phenols, resulting in a decrease or increase, respectively, in the reported rate constants [134]. The use of the DPPH assay as a reliable method for measuring antiradical activity is compromised due to its chemical limitations. Under acidic circumstances, the reducing capacity of antioxidant compounds may be hindered as a result of protonation. Conversely, under basic conditions, the dissociation of protons from phenolic compounds would augment the reducing ability of a sample. Furthermore, a recent investigation revealed that the outcomes pertaining to the impact of flavonoids in the DPPH test were contingent upon the Bors criterion. Conversely, the association between the chemical structure and activity in the ABTS assay remained ambiguous. The ABTS test showed elevated findings only for phenolic acids with pyrogallol structures, while the DPPH assay primarily relied on the quantity of hydroxyl (OH) groups [135]. This could explain the differentiated results for measuring the Antioxidant capacity of the beverages before and after the fermentation with ABTS and DPPH assays. Stratil and colleagues noted that the radical cation of ABTS (ABTS^•+^) is the most reactive and responds to the highest number of hydroxyl groups of the phenolic compounds of wines [123].

The antioxidant activity of samples A, B, and C decreased significantly (*p* < 0.05) for both ABTS and DPPH assays but remained relatively high after 30 days of cooling at 4 °C. The mean IC_50_ values for ABTS were 24.39–25.27, from 20.74 to 22.84 after and before refrigeration, and between 37.50 and 42.05 from 32.39 to 35.40 for DPPH, respectively. The differences between the three samples were negligible, justified by the respective elimination of differences in total phenolic and flavonoid values.

### 3.9. α-Amylase and α-Glucosidase Inhibition

Incorporating starch hydrolase inhibitors into the diet has retard glucose absorption by inhibiting α-amylase and α-glucosidase. These two enzymes are essential in the breakdown of carbohydrates, which are present in the small intestinal brush border. Therefore, inhibiting these enzymes’ activity will reduce the glucose absorption rate into the blood, thereby reducing the physiological glucose levels.

**Figure 6:**
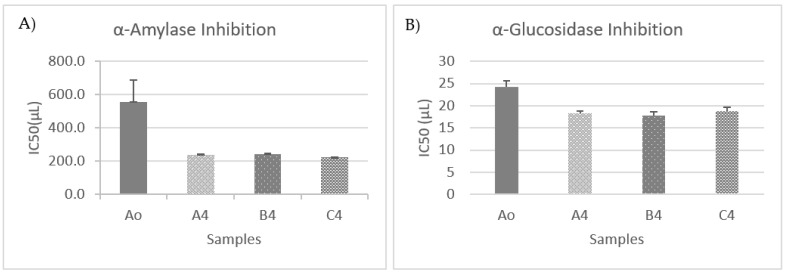
(**A**) α-Amylase inhibition from OMTWH kombucha samples before (A0) and after (A4, B4, C4) fermentation. Results are expressed as mean ± standard deviation in μL required for 50% inhibition of a-amylase of three independent measurements. (**Β**) α-Glucosidase inhibition from OMTWH kombucha samples before (A0) and after (A4, B4, C4) fermentation. Results are expressed as mean ± standard deviation in μL required for 50% inhibition of a-glucosidase of three independent measurements.

The α-amylase inhibitory activities are shown in Figure 6A. Overall, the α-amylase inhibitory activities of OMTWH’s IC_50_ values were 543 ± 44 μL before the fermentation process and 236 ± 7.0, 244 ± 4.0, and 222 ± 1.0 μL for samples A, B, and C after the fermentation.

The α-glucosidase inhibitory activities are shown in Figure 6B. The α-glucosidase inhibitory activities of OMTWH’s IC_50_ values were 24.4 ± 1.2 μL before fermentation and 18.3 ± 1.1, 17.8 ± 0.9, 18.8 ± 0.8 μL for samples A, B, and C after the fermentation.

In addition, the IC_50_ values in both cases exhibited statistically significant decreases (*p* < 0.05); therefore, the α-amylase and α-glucosidase inhibitory activities increased after fermentation.

Therefore, results revealed that OMTWH had a potent inhibitory activity of α-amylase and α-glucosidase, and OMTWH fermented with the kombucha consortium exhibited even higher inhibition.

The results are in agreement with previous studies, where it was observed that the α-amylase inhibitory activity had increased with the fermentation process, and this could have been due to the increase in the total phenolic content as a result of the fermentation process [136,137]. In vitro inhibition of α-amylase and α-glucosidase was also noticed [16,138] in green, black, and oolong tea kombuchas.

The IC_50_ values of α-amylase and α-glucosidase inhibitions showed strong negative correlations (Pearson correlation α-amylase = −0.870, Pearson correlation α-glucosidase = −0.914, *p* < 0.01) with the values of total phenolic compounds during fermentation. That indicates an increase in the inhibition of α-amylase and α-glucosidase with the rise in the number of phenolic compounds in the samples. Good correlation (r > 0.90, *p* < 0.01) between the total phenolic content and the inhibition activities of α-glucosidase and α-amylase were also observed [35]. This result is consistent with another study, finding that the inhibition of phenolic extracts from oats to α-glucosidase and α-amylase is dose-dependent [139].

Furthermore, previous scientific studies demonstrated that plant-derived extracts (including infusions) and phytochemicals (polyphenols) are potential alternatives to synthetic inhibitors of α-amylase and α-glucosidase [140,141]. In addition, phenolic compounds are considered good regulators for carbohydrate and lipid metabolism through the inhibition activities of α-glucosidase and α-amylase due to their chelating, structure-altering, and biological function-limiting capacity to the enzymes [142,143].

The inhibition of α-amylase slows the liberation of maltose from starch, delaying the conversion to glucose and decreasing postprandial plasma glucose levels. Whereas the inhibition of α-glucosidase retards the release of D-glucose from oligosaccharides and disaccharides, delaying glucose absorption and lowering the postprandial plasma glucose levels [144]. Therefore, the use of OMTWH beverage fermented with the Kombucha consortium as an anti-diabetic agent might be expected.

Further in vivo and clinical studies are warranted to demonstrate their therapeutic potential to be relevant to human health.

### 3.10. Cholinesterase Inhibition Assays

**Figure 7:**
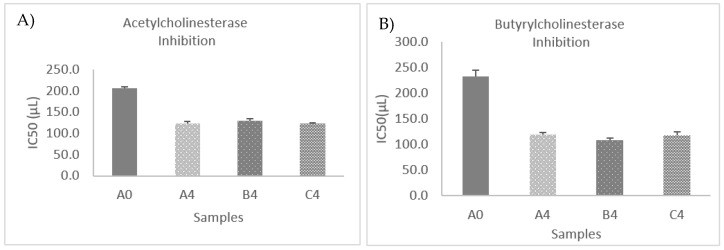
(**A**) Ache Inhibition from OMTWH kombucha samples before (A0) and after (A4, B4, C4) fermentation. Results are expressed as mean ± standard deviation in μL required for 50% inhibition of acetylcholinesterase of three independent measurements. (**B**) BChe Inhibition from OMTWH kombucha samples before (A0) and after (A4, B4, C4) fermentation. Results are expressed as mean ± standard deviation in μL required for 50% inhibition of butyrylcholinesterase of three independent measurements.

The AChE and BChE inhibitory activities are shown in Figure 7A,B. The IC_50_ values in both cases exhibited statistically significant decreases (*p* < 0.05); therefore, AChE and BChE inhibitory activities increased after fermentation. In a recent research, it was shown that the anti-acetylcholinesterase activity exhibited an increase when subjected to fermentation. This increase resulted in about an eight-fold higher value in both the ethyl acetate and aqueous extracts of B. tournefortii, in comparison with the unfermented samples [145]. Results suggested that kombucha fermentation may generate new active metabolites against AChE in the extracts.

Alzheimer’s disease (AD) is the most common form of dementia. AD begins as a deficiency of acetylcholine (ACh) inhibitors. Acetylcholinesterase (AChE) and butyrylcholinesterase (BChE) cause ACh hydrolysis, a significant neurotransmitter responsible for the brain’s cognitive functions. Inhibition of cholinesterase enzymes is a common strategy for the treatment of Alzheimer’s disease.

The Anticholinesterase activity in OMTWH before fermentation can be attributed to *Sideritis scardica* and thyme honey. Studies indicated that various extracts of *Sideritis* species showed moderate cholinesterase inhibitory activity [146,147,148,149]. Furthermore, there are studies that suggest anti-AChE and anti-BChE activities in honey. The analyzed kinds of honey in a recent study showed various AChE and BChE inhibition [150]; specifically, the highest increase in AChE inhibition (21.17%) was observed for thyme honey. Regarding BChE activity inhibition, the highest significant result was recorded for goldenrod honey (33.89%).

The IC_50_ values of AChE and BChE inhibitions showed strong negative correlations (Pearson correlation AChE = −0.890, Pearson correlation BChE = −0.931, *p* < 0.01) with the values of total phenolic compounds before and after fermentation. That indicates an increase in the inhibition of AChE and BChE with the rise in the number of phenolic compounds in the samples. The above is in accordance with studies that suggested a high correlation between TPC and AChE inhibition activity [151,152].

Furthermore, another research investigation revealed a statistically significant positive link (*p* < 0.001) between the overall phenolic content and both the antioxidant activity and anticholinesterase capability of Elatostema papillosum leaves [153]. With respect to the anti-dementia activity of kiwifruits in a previous study, AChE inhibitory activity had the most significant correlation with TP content (r^2^ = 0.93) [154]. In contrast, the correlation coefficients of AChE and TF content among the kiwifruits was r^2^ = 0.12. BChE activity was not significantly correlated with TP (r^2^ = 0.23) content. However, the activity was significantly correlated with TF (r^2^ = 0.57). In a recent study, the high anti-ChE inhibitory activity of kinds of honey was linked with high polyphenolic contents [150], while a previous study showed that a high anti-AChE activity of studied kinds of honey was correlated with their high antioxidant activities, and consequently with the high TPC in the studied kinds of honey [155].

Furthermore, Zaidi et al. discovered noteworthy inhibitory effects on acetylcholinesterase (AChE) in a total of 31 diverse types of Algerian honey. The honey samples were found to possess a considerable quantity of total phenolic content (TPC). Furthermore, the investigators observed a statistically significant and strong correlation between the anti-acetylcholinesterase (anti-AChE) activities and total phenolic content (TPC) levels in the honey samples that were subjected to examination [156]. Similarly, a recent investigation [157] conducted on a total of 47 distinct samples of Polish honey has shown further evidence supporting a positive correlation between the activity of anticholinesterase enzymes (anti-AChE and anti-BChE) and the total phenolic content (TPC).

## 4. Conclusions

This study presents a novel beverage as well as its physicochemical analysis and an evaluation of its functional properties.

The beverage was prepared with Olympus Mountain tea (*Sideritis scardica*), sweetened with thyme honey, and fermented by kombucha culture. Four days of fermentation appeared sufficient to improve the nutritional and functional characteristics of the beverage.

The total counts of bacteria and yeast increased daily to 4.7 × 10^6^ and 2.9 × 10^6^ CFU/mL, respectively, on fermentation’s 4th and final day. The increase was indicative of the ability of the OMTWH to act as a suitable substrate to sustain the proliferation of the microorganisms of the kombucha symbiotic culture. Due to the kombucha fermentation, total sugars decreased, and ethanol and TA increased with acetic and gluconic as the dominant acids. The decrease in pH values followed the increase in acidity. In our case, optimal consuming acidity was obtained for less than four days of fermentation. The results showed that mountain tea with honey is a suitable substrate, respectively, with tea and sugar, for microbial growth in symbiotic kombucha culture fermentation. Moreover, total phenolic compounds, vitamin C, and vitamin B-complex content increased after fermentation. The IC_50_ values of antioxidants for ABTS decreased after fermentation, which denotes increasing antioxidant activity. The strong negative correlation between total phenolic content and ABTS IC_50_ values (Pearson Correlation = −0.837, *p* < 0.01) further confirmed this point.

Results revealed that the unfermented OMTWH had a potent inhibitory activity of α-amylase, α-glucosidase, AChE, and BChE; OMTWH fermented with a kombucha consortium exhibited even higher inhibition. Therefore, kombucha fermentation can transform OMTWH into a novel beverage with enhanced functional properties.

## Figures and Tables

**Table 1:** Chromatic parameters of OMTWH before and after fermentation.

	A0	A4	B4	C4
L*	60.6 ± 0.59 ^a,1^	66.1 ± 0.51 ^b^	66.4 ± 0.49 ^b^	67.1 ± 0.53 ^b^
a*	0.4 ± 0.02 ^c^	−4.1 ± 0.24 ^d^	−4.3± 0.25 ^d^	−3.8 ± 0.21 ^d^
b*	29.3 ± 0.64 ^e^	22.3 ± 0.53 ^f^	22.4 ± 0.56 ^f^	21.4 ± 0.61 ^f^
ΔΕ*	-	10.0 ± 0.1 ^g^	10.2 ± 0.1 ^g^	11.1 ± 0.1 ^g^

^1^: Different letters in each row indicate significant difference (*p* < 0.05).

**Table 2:** Content of vitamins of the B-complex before and after fermentation.

Samples	Vitamins (μg/mL) ^1^				
	B1	B6	B12	B2	B7
A0	0.15 ± 0.01 ^a^	0.59 ± 0.02 ^a^	1.78 ± 0.04 ^a^	4.37 ± 0.09 ^a^	2.79 ± 0.09 ^a^
A4	1.74 ± 0.04 ^b^	2.71 ± 0.08 ^b^	2.18 ± 0.06 ^b^	5.47 ± 0.16 ^b^	3.42 ± 0.11 ^b^
B4	2.64 ± 0.08 ^c^	3.12 ± 0.09 ^c^	3.30 ± 0.08 ^c^	5.39 ± 0.13 ^b^	3.27 ± 0.10 ^b^
C4	2.59 ± 0.06 ^c^	2.99 ± 0.09 ^c^	3.46 ± 0.10 ^c^	5.34 ± 0.12 ^b^	3.16 ± 0.12 ^b^

^1^: Results are presented as mean ± standard deviation of three independent measurements. Different letters in each column indicate significant differences.

## Data Availability

The data used to support the findings of this study can be made available by the corresponding author upon request.
